# Metasurfaces for Advanced Sensing and Diagnostics

**DOI:** 10.3390/s19020355

**Published:** 2019-01-16

**Authors:** Luigi La Spada

**Affiliations:** School of Engineering and the Built Environment, Edinburgh Napier University, 10 Colinton Rd, Edinburgh EH10 5DT, UK; l.laspada@napier.ac.uk; Tel.: +44-131-455-2696

**Keywords:** MetaSurface, modeling, design, sensors, cancer detection, glucose measurements, medical diagnostics

## Abstract

Interest in sensors and their applications is rapidly evolving, mainly driven by the huge demand of technologies whose ultimate purpose is to improve and enhance health and safety. Different electromagnetic technologies have been recently used and achieved good performances. Despite the plethora of literature, limitations are still present: limited response control, narrow bandwidth, and large dimensions. MetaSurfaces, artificial 2D materials with peculiar electromagnetic properties, can help to overcome such issues. In this paper, a generic tool to model, design, and manufacture MetaSurface sensors is developed. First, their properties are evaluated in terms of impedance and constitutive parameters. Then, they are linked to the structure physical dimensions. Finally, the proposed method is applied to realize devices for advanced sensing and medical diagnostic applications: glucose measurements, cancer stage detection, water content recognition, and blood oxygen level analysis. The proposed method paves a new way to realize sensors and control their properties at will. Most importantly, it has great potential to be used for many other practical applications, beyond sensing and diagnostics.

## 1. Introduction

Recently, there has been an increased interest in exploiting electromagnetic fields and waves to develop advanced sensing and medical diagnostics devices. Tissue diseases typically induce structural, biochemical, and mechanical changes, implying significant variations in their electromagnetic properties. The main aim of an electromagnetic sensor is to reveal such differences [1]: the disease generates change(s) in the electromagnetic properties of the sample (namely, refractive index or absorption), the sensor detects such modifications in terms of frequency position, magnitude, and bandwidth on its output signal. To this regard, micro- and/or nano- structures have been used as sensing platforms with applications in medicine, biology, environment, and safety [2]. Considering the wide range of materials and samples to investigate, electromagnetic sensors possess advantages compared to traditional technologies: speed, low-cost, high sensitivity, and selectivity, as well as label-free analysis [3]. 

In the literature, a huge variety of electromagnetic sensors exists, namely: interferometers [4], waveguides, and gratins [5], Surface Plasmon Resonance (SPR)-based structures [6], cavities/resonators [7], and the most recent all-dielectric metamaterials [8]. They show some of the above-mentioned advantages; at the same time, they possess some limitations and drawbacks [9,10]: electrically large structures, high losses, dispersive behavior, polarization dependence, and difficult to manufacture. In this scenario, a relevant role is played by MetaSurfaces: the 2D version of metamaterials. MetaSurfaces are artificial materials with exotic electromagnetic characteristics. They are composed by metallic/dielectric inclusions [11,12,13], whose dimensions and spatial periodicity are much smaller compared to the operative wavelength, typically arranged in array configuration [14,15,16]. Thanks to their inclusions (geometry, material) and arrangements, MetaSurfaces exhibit unprecedented properties not easily found in traditional materials and/or existing technology [17,18,19,20,21,22,23]. In the past, such properties have been widely used and implemented to enhance devices such as: antennas [24], absorbers [25], guiding structures [26], polarizers and modulators [27], lenses for imaging systems [28] and cloaking devices [29]. 

Despite the huge advancement, intrinsic fundamental limitations are still present in such technologies: limited control on their response; highly confined near-field; narrow bandwidth; manufactured for limited and specific geometries, source, and polarization dependent. To date, these are still unsolved problems. 

To overcome such issues, in this paper, a generic and versatile tool able to control and manipulate MetaSurface properties at will, will be developed. The aim is manifold:

1. control simultaneously all the MetaSurface properties: amplitude, phase and bandwidth;
2. exploiting electromagnetic characteristics, typically considered detrimental for other devices (such as near-field, narrow bandwidth, and so on) to develop MetaSurface structures with high selectivity and sensibility;
3. use them for advance sensing and diagnostics platforms.

In the first part of the paper (Materials and Methods), the proposed modeling and design approach is presented. The aim is to control MetaSurface electromagnetic properties, by linking them to its physical dimensions. This will permit to manufacture the structure for the application required. To this regard, in the second part (Results and Discussion), the paper will demonstrate how to use the new approach to enhance existing devices in the field of advanced sensing and medical diagnostics. 

## 2. Materials and Methods: A Generic Tool to Realize Arbitrary Shape Metasurfaces

In this section, we have two main objectives:

1. Modeling: link the propagation properties of the impinging electromagnetic wave (electric (E) and magnetic (H) components) with the structure impedance distribution *Z*(r).
2. Design: obtain the relation between the Impedance *Z*(r) and the MetaSurfaces physical properties: inclusions dimensions (length *l*, width *w*, gap *g,* and thickness *t*), substrate thickness *d*, and spatial periodicity Λ.

**Figure 1:**
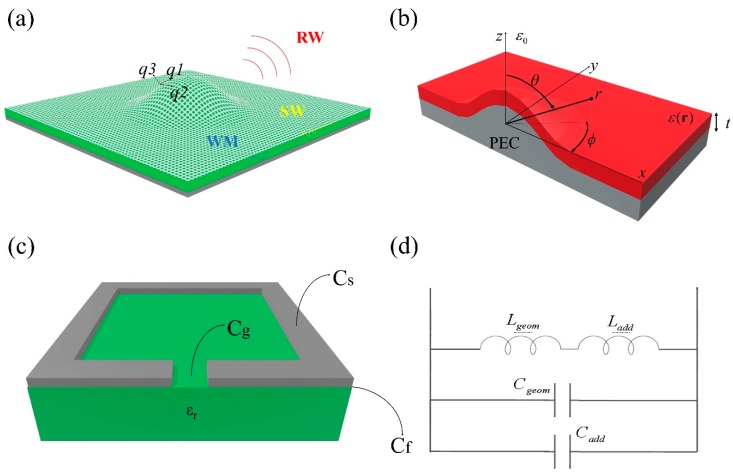
(**a**) Three-dimensional curvilinear MetaSurface structure: ground plane (dark grey), homogeneous dielectric substrate (green) of permittivity ε_r_, and thickness *d*, MetaSurface (light grey) placed on top of it. (**b**) Equivalent non-homogeneous model of the structure in (**a**): MetaSurface + substrate is represented by a layer of non-homogeneous permittivity ε(**r**), magnetic permeability µ_0_, and thickness *t*, the metallic object underneath is a Perfect Electric Conductor (PEC). (**c**) MetaSurface unit-cell: metallic square-shape inclusion (grey) deposited on the homogeneous dielectric substrate. The physical dimensions of the metallic particles are: side length (l), gap (g), strip width *w*, thickness (t.) (**d**) Equivalent circuit model for the metallic square-particle of the unit cell in (**c**): the circuit impedance contains both geometrical terms (C_tot_, L_tot_) and additional terms (C_add_, L_add_).

Figure 1a shows the geometry of a generic curvilinear MetaSurface formed by (metallic/dielectric) patches, printed on a grounded substrate. The substrate slab has thickness *d*, permittivity ε_r_, and permeability µ_r_ = µ_0_ (µ_0_ magnetic permeability of free space). The top layer is free-space with permittivity ε_0_ and permeability µ_0_, from where the electromagnetic wave impinges with an incident angle θ_i_. It can assume different forms at the interface: reflected, transmitted, and/or absorbed. Any alteration to the boundary conditions causes changes in the wave propagation characteristics. Such modifications are mainly due to the geometry and the electromagnetic parameters of the materials at the considered interface [30]. To this regard, we can classify the wave propagation as radiated, guided-modes, and surface-waves, labeled RW, WM, and SW, respectively (Figure 1a). 

In the past, to control such waves, huge efforts have been focused on ultrathin metallic MetaSurfaces and/or their dielectric counterparts [31,32,33,34]. It has been recently demonstrated in [35] that electrically thin MetaSurfaces are not able to fully manipulate waves. Since the aim of this paper is to achieve a full control of all the wave propagation characteristics, the MetaSurface should have finite (yet small) thickness. This permits to model it as a slab having non-homogeneous electric permittivity ε(r) and/or magnetic permeability μ(r). In this paper, we consider the MetaSurface having non-homogenous permittivity ε(r) and homogeneous permeability µ_r_, as depicted in Figure 1b.

To relate both electric E and magnetic H wave components and its characteristics (amplitude, phase, and bandwidth) with the impedance *Z*(r) of the MetaSurface (point 1), it is crucial to solve the appropriate Helmholtz wave equations. Its solutions for homogeneous materials are known in literature [36]. On the other hand, for non-homogeneous media (Figure 1b), the equations and the related solutions became more complex:

(1a) $$\nabla^{2}E+\nabla \left[E\cdot \nabla \log\varepsilon (r)\right]+\omega^{2}\mu_{r}\varepsilon (r)E=0$$

(1b) $$\nabla^{2}H+\frac{1}{\varepsilon (r)}\left[\nabla \varepsilon (r)\right]\times \left(\nabla \times H\right)+\omega^{2}\mu_{r}\varepsilon (r)H=0$$

where the constitutive parameters (*ε*(**r**), *µ*_r_) are linked to its impedance as follows [37]:

(2) $$Z(r)=\sqrt{\frac{\mu_{r}}{\varepsilon (r)}}$$

To relate the impedance distribution in (2) with the MetaSurface geometry and physical dimensions (point 2), let us consider the structure shown in Figure 1c: a metallic square-shape particle deposited on a homogeneous dielectric substrate. To satisfy the impedance distribution in (2) we need to carefully design the following: 

(a) **The unit-cell:** When a wave is impinging on metallic (dielectric) patches, both electric € and magnetic (H) fields are excited [38]: a time-varying magnetic (H) component perpendicular to the surface; and electric (E) components in the gap, within the substrate and along the surface. To each electric and/or magnetic components corresponds related induced density currents, namely: electric currents J_e_ = nxH, equivalent magnetic J_m_ = −nxE, and electric displacement J_d_ = ε(r)E. According to the dual principle [37], the structure of Figure 1c will have an electric (magnetic) resonance mode. It means that transverse electric (magnetic) currents will flow with high intensity along the metallic (dielectric) layer of the patch. At the same time, transverse magnetic (electric) currents will be very weak in the same region [39]. Knowing the electromagnetic configuration of the patch of Figure 1c, its equivalent circuit impedance can be easily evaluated as [37]:

(3) $$Z=\frac{\int_{a}^{b}E(r)•dl}{\oint_{C}H(r)•dl}$$

where ***l*** is the line chosen element, (a, b) the electric potential points and C the closed magnetic loop. 

By using (3), the patch of Figure 1c can be represented by a shunt Impedance *Z_unit-cell_* composed by a (real part), the Resistance *R*(**J_s_**); and an imaginary part, the Reactance X_MS_ of inductive X_L_(**J_m_**) = *jωL* or capacitive X_C_(**J_d_**) = 1/*jωC* nature for metallic or dielectric patches, respectively. Complete expressions for the parallel or series impedance are reported in Table 1. 

Both capacitive *C_tot_* and inductive *L_tot_* terms for the equivalent circuit model of Figure 1d, can be evaluated as a function of the geometry and size of the inclusions, by using formulas coming from electrostatics [40] and magnetostatics [41], respectively:

(4) $$C=\frac{{∯}_{S}D\cdot dS}{\int_{a}^{b}E\cdot dl},L=\frac{{∯}_{S}B\cdot dS}{\oint_{C}H\cdot dl}$$

where S is the surface element. 

For the patch of Figure 1c, the total capacitance C_tot_ can be described as the superimposition of the gap, fringing, and surface capacitance, respectively [42]: C_tot_ = C_g_ + C_f_ + C_s_. The total inductance L_tot_ is, instead, composed by self and mutual inductance, respectively [43]: L_tot_ = L_self_−M. Complete expressions for capacitance and inductance can be found in Table 1 and in [42].

(b) **The thickness:** In [39] it has been demonstrated that a single metallic (dielectric) ultrathin MetaSurface has an inductive (capacitive) behavior, which only introduce a discontinuity on the transverse magnetic (electric) field. To fully and properly satisfy all the boundary conditions, a discontinuity of the transverse electric (magnetic) field is necessary, by introducing an impedance element in series. The presence of both parallel and series circuit elements in the form of П- or T- network determines a discontinuity for both electric and magnetic components of the wave, allowing full control of its properties. 

The patch of Figure 1c is deposited on a dielectric substrate of finite thickness *d*. In this case electric displacement currents **J_d_** = jωε(**r**)**E** arise, equivalent to a capacitive reactance X_C_(**J_d_**) = 1/jω*C(ε_sub_)* in series [49]. At infrared and optical frequencies, additional energy is stored within the particle [50]: The inductive inertia of the electrons oscillating in the metal (**J_m-add_**) and the electron potential energy (**J_d-add_**) created by separate charges within the metal, determingn an inductive X_L_(**J_m-add_**) = jω*L_add_*, or capacitive X_C_(**J_d-add_**) = 1/jω*C_add_* reactance, respectively. Complete expressions for such elements are reported in Table 2 and in [42].

(c) **The coupling phenomena:** In previous works it has been generally assumed that electromagnetic coupling between adjacent unit-cells can be neglected [39]. On the contrary, it plays an important role for the full control of the wave properties, especially in terms of highly confined signal, and multi/broad -band behavior. In presence of more than one structure, we need to consider the mutual effects among adjacent unit-cells: inductive magnetic flux *ψ_m_*(**B**) and/or displacement electric charges *Q_e_*(**D**), respectively. Adjacent structures can result magnetically and/or electrically coupled, represented in the circuit-model by the mutual inductance *Z_M_*(*ψ_m_*) = L_2_/L_1_ and/or mutual capacitance *Z_C_*(*Q_e_*) = C_2_/C_1_, respectively. Complete expressions for the mutual terms can be found in Table 3. 

## 3. Results and Discussion

We now use the approach described in the previous section to realize MetaSurface-based structures for advanced sensing and diagnostics applications. The MetaSurface (the sensor) consists of metallic inclusions arranged in an array configuration, whose frequency response is modified by the surrounding environment (the biological sample) changes. 

**Figure 2:**
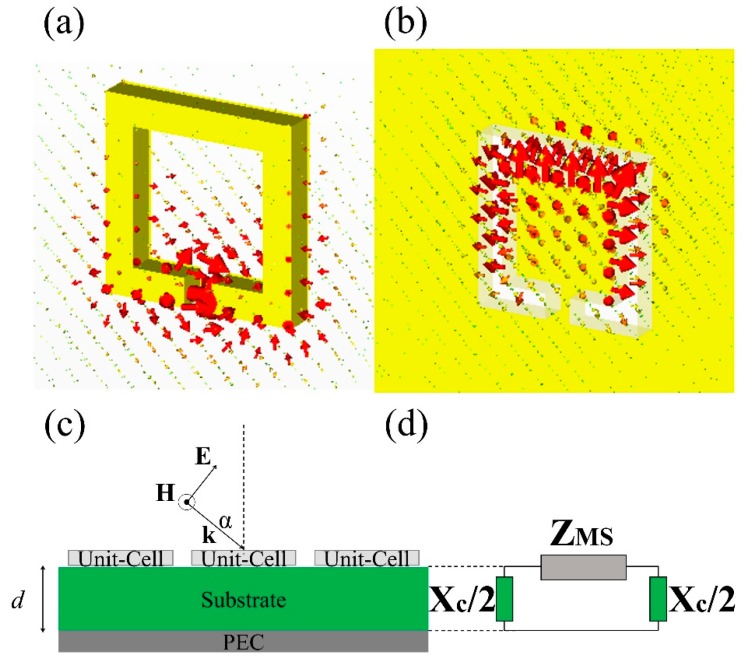
(**a**) Split Ring Resonator (SRR). The electric component (red arrows) is localized in specific spots: gap, substrate, and surface. The high electromagnetic field concentration makes SRRs more suitable to develop high sensitivity platforms. (**b**) Complementary SRR and CSRR configurations possess both a greater sensing area and most importantly pencil-like radiation patterns. It is possible to design sensors with high selectivity for the recognition of specific compounds, by tuning its frequency(ies) with the absorption peak(s) of the sample. (**c**) Propagation model (side-view): The MetaSurface is composed by the square-shape unit cells (light grey) of Figure 1c arranged in array disposition with equal periodicity, Λ. The supporting layer is a homogeneous dielectric slab of permittivity (ε_r_) and thickness (d). The substrate is grounded by a metallic plate at the bottom, acting as PEC. (**d**) Equivalent transmission-line model of structure in Figure 2c. We consider each layer as a section of the transmission line, each of one characterized by their impedances: Z_MS_ is the impedance of the MetaSurface structure, considering both Z_series/parallel_ (for SRR/CSRR) and Z_mutual_ (mutual capacitive/inductive coupling phenomena). X_c_ is the thickness substrate impedance. The PEC layer is represented by a short circuit Z_PEC_ = 0.

Two main configurations are here considered in realizing MetaSurfaces: Split-Ring-Resonator (SRR) and Complementary-SRR (CSRR), shown in Figure 2a,b, respectively; where the electric (magnetic) component distribution is depicted by red arrows. The arrangement in Figure 2c shows that the plane wave impinges on the planar array of metallic inclusions along the direction, k, with an angle, α, and the magnetic (H) component parallel to the surface. By using formulas (1)–(4), the structure Impedance is:

(5) $$Z_{structure}=Z_{MS}\sqrt{1-\frac{\sin^{2}\alpha }{X_{C}}}\tan\left(\frac{\omega }{c}Z_{MS}d\right)$$

where *Z_MS_* can assume the value of *Z_series_* or *Z_parallel_* of Table 1, as a function of the unit-cell used SRR or CSRR, respectively. For both cases, SRR and CSRR, three sensing areas can be identified represented by the related capacitive terms described in Table 1, respectively: the gap C_g_(*g*, *w*, *t*), the surface C_s_(l, w, g, t), and the fringing C_f_(*w*, *g*). The aim is to evaluate the expression of the mentioned capacitive terms and consequently find out the correct dimensions to realize MetaSurfaces accordingly to the required applications.

**Figure 3:**
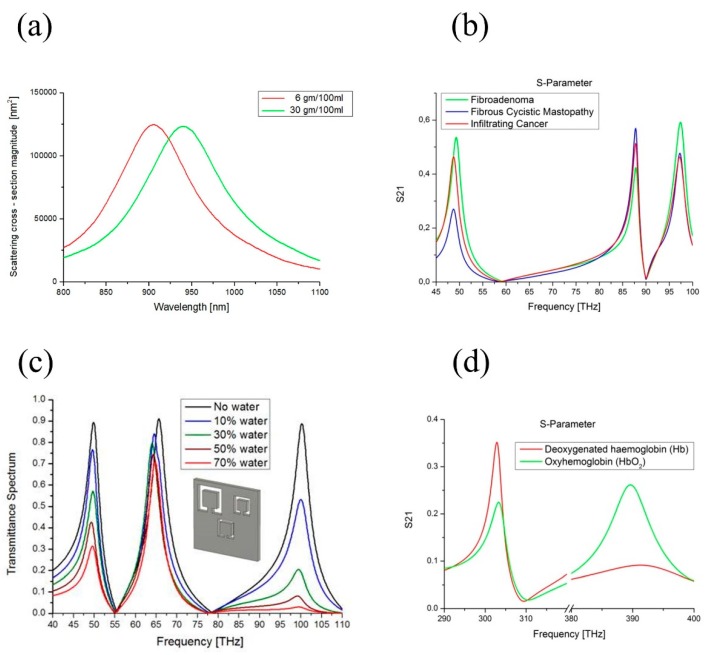
(**a**) Sugar level detection. The sensing platform consist in an array of rectangular metallic inclusions designed to operate in the infrared range (800 nm–1000 nm), where the refractive indices of sucrose, sodium chloride, glucose, and caster sugar solutions have been measured for different density as a function of their concentration. The shift in the resonant of the reflection coefficient is related to such concentration variations. In the example, the substance is glucose varying from low concentration in red (6 gm/100 ml, refractive index *n* = 1.34) to high concentration green (30 gm/100 ml, refractive index *n* = 1.36), corresponding to a shift of more 50 nm in the reflection peak for a difference in the refractive index equals to Δn = 0.02. (**b**) Cancer stage recognition. The sensor consists of a planar CSRR made of circular inclusions. The resonant frequencies of the sensor (50, 87, and 99 THz) are designed to coincide with the proteins and lipids spectral absorption characteristics of three breast sample tissues: infiltrating cancer (red), fibrous-cystic mastopathy (blue), and fibroadenoma (green). The transmission coefficient peaks of the CSRR structure change both magnitude and amplitude width, accordingly to the molecular bonds absorption rate of the considered tissues at such frequencies. (**c**) Water content detection. The sensing platform consist of a planar metallic CSRR composed by square-shape inclusions. It presents multiple resonant frequencies (50, 66, and 100 THz), tuned to the ones of water molecule vibrational modes. In black is the sensor response without the biological compound. The sensor allows recognizing the presence of water in biological tissues for different percentages: 10%, 30%, 50%, and 70%. The changes in the transmission coefficient magnitude and amplitude width are related only to the absorption rate of the hydrogen bonds of water molecules. All the transmission peaks significantly absorb in a different way, in line with the absorption behavior of water at such frequencies. (**d**) Oxygen levels in human bloodstream. The structure consists of a single square CSRR particle, presenting multiple resonant frequencies, coincident with the infrared absorption frequencies of the Oxyhemoglobin (HbO_2_) and deoxygenated hemoglobin (Hb) in human blood: 315 and 400 THz. The transmission spectrum of the CSRR changes both magnitude and amplitude width proportionally to the sample absorption rates: HbO_2_ has its lower absorption at 660 nm (400 THz) compared to Hb having its higher absorption at 940 nm (315 THz).

As previously mentioned, biological tissue properties and their frequency response are the results of the interaction between the electromagnetic radiation and their constituents at molecular and cellular level [51]. Any alterations imply significant changes in the related electromagnetic properties, leading to frequency (blue or red) shift or amplitude variation in the output signal of the MetaSurface sensor (Figure 3).

The shift of the resonant frequency is related to the real part of the sample refractive index, while the enlargement of the amplitude is related to the imaginary part (losses): its dissipative behavior. By evaluating the resonant properties of the MetaSurface platform (peak position, amplitude, and bandwidth), the biological sample under study can be traced. In fact, through the frequency position of the resonant dip, it is possible to distinguish the substance that we are looking for, as shown in Figure 3a,b. On the contrary, the resonance magnitude and its bandwidth vary depending on the amount of radiation absorbed, which is related to the concentration of the sample under study, as reported in Figure 3c,d. Therefore, to detect such alterations/phenomena, two kind of measurement setups can be used:

1. Refractive index measurements: the sensor, without any material has a specific resonant frequency. Once the material to study is placed in contact, the overall device is illuminated by an electromagnetic wave. The sample properties are revealed by changes in the resonant characteristics such as wavelength position, magnitude and bandwidth.
2. Absorption measurements: the sensor is placed not in direct contact with the biological sample. In this configuration, the electromagnetic absorption phenomena of the sample play a crucial role. The absorption measurement is revealed by the changes in the transmission coefficient magnitude and bandwidth, while the resonant wavelength position doesn’t change.

Further details regarding the use of MetaSurfaces as sensors for refractive index and absorption measurements are revised below for the following sensing and medical diagnostic applications: molecule detection [52], biosensing [53] and microfluidic [54].

### 3.1. Cavity/Resonator Modes for Molecule Detection

The refractive index of solutions increases with the increasing of the chemical species concentration. Therefore, it would be possible to sense the presence of either organic or inorganic compounds. For an accurate detection of such substances, a huge electric field enhancement can be obtained by exploiting cavity/resonator modes (gap capacitance *C_g_* [44] of Table 1).

A practical application of the proposed approach can be found in [55,56,57], where a planar MetaSurface sensor has been studied for glucose detection. The highly sensitive and selective sugar detection sensor was designed and realized by using nano-antennas technology. The strongly localized and enhanced transmission obtained in the cavity gap of the nano-antennas highly increase the molecular absorption to allow the selective detection of D-glucose and sucrose at low concentrations (Figure 3a). Thanks to its high sensitivity (30k nm/RIU [58]), this device holds the potential for further practical applications to be fabricated as a lab-on-a-chip device. 

In addition, microorganisms have frequently been explored in sensing applications due to the comparable size of a microorganism to the size of the unit-cell gaps. Slot antenna arrays were used to demonstrate individual yeast cell detection [59,60], where yeast cells caused a huge shift in the peak resonant frequency as a function of the micro-organism’s shapes [61]. The sensor can be widely used for monitoring the cell status during natural growth or after treatment. Since this sensor is extremely sensitive to the number of the external cells, it be also used in label-free measurements of cell apoptosis. In a similar way SRRs have been used for fast and accurate detection of fungi and bacteria [61] like Escherichia coli. Extremely small amounts of the microorganisms were detected, as their sizes were on the same scale as the micro-gaps. As the excited electric field was very high, the intensity of the water absorption peaks was not so strong, making this sensor usable in liquid sensing, with the most sensitive area locating at the gap of the SRR, overcoming the classical issues of free-space sensing techniques.

Food security and safety are recently receiving huge attention [62,63,64], especially due to the use of external addictions, such as pesticide and antibiotics. They became a great concern because of the harmful effects they can have on human health and the environment. A MetaSurface consisting of square/circle-shaped slits has been used for kanamycin sulfate [65] and tetracycline hydrochloride (TCH) [66] detection. High sensitivity had been reached (600 nm/RIU [67]) with the minimal detectable concentration, due to the existence of huge electric field amplification at the gaps. 

In [68] an SRR structure has been developed for the detection of pesticide like chlorpyrifos-methyl in vegetables. SRR/cavity-like structures can be used for refractive index measurements and the detection of (non-)transgenic products [69]. Corrugated metallic surfaces consisting of a linear array of subwavelength grooves have been used [70] to detect lactose. A hybrid planar plasmonic waveguide composed of a subwavelength plastic ribbon [71] and a diffraction grating was also employed to detect analytes in powdered form (450 nm/RIU).

### 3.2. Wave-Guide Modes for Biosensing

Planar SRR structure arrays and substrate wave-guide modes (fringing capacitance *C_f_* [45] in Table 1) have been theoretically studied [72] and fabricated [73] for the detection of liquid samples. The presence of water produces changes in biological material refractive index values: both real and imaginary parts [74]. Tumors possess a significantly higher water content compared to healthy tissues [75]. Therefore, both permittivity ε and conductivity σ of tumors are higher than those of a normal tissue. This is valid not only at microwaves but also at higher (infrared) frequencies. Such property turns out to be a useful tool for tissue water content [76], tumors [77], and stage diseases [78] detection, Figure 3b.

Hematological diseases induce structural, biochemical and mechanical changes in red blood cells (RBCs) [79]. The structural variations imply significant changes in cell electromagnetic properties. The refractive indices of different kind of RBCs differ in their real and imaginary part [80]. For this reason, the presented method has been applied to realize the sensor in [81]: the MetaSurface sensor changes its position, as a function of the different RBCs structural modifications, showing the sensor capability to distinguish healthy RBCs from specific malaria diseases, such as Schizont and Trophozoite.

Ethanol-water mixture and aqueous solution of NaCl with different concentrations were investigated in [82] and it has been shown that the peak value (in terms of frequency and bandwidth), near the resonant region, depends linearly on the solution concentration. It comes from the variation of dielectric environment close to the interface between the MetaSurface and the aqueous sample (Figure 3c).

Glycerol concentration measurement is crucial for several application fields, such as biomedical engineering, medicine and biofuels fabrication. Evaluating levels of glycerol, it is a useful parameter to evaluate in various pathological conditions [83]. Glycerol measurement in aqueous solutions is not simple because its permittivity varies very little by changing its concentration. Table 1 has been used to design CSRR-based sensors in [84] for the detection of glycerol-water mixtures (7000 nm/RIU [85]).

Further remarkable examples of such sensing technique can be found in hybrid solutions of metallic MetaSurface and substrates made of Near-Zero-Index (NZI) materials [86] to measure the refractive index of polar liquid solutions and polymers. Such materials can strongly attenuate, trap, and enhance the incident wave into the thin substrate layers. The resonance shift is mainly caused by the real dielectric constants of the liquid solution. Based on this idea, in [87], an array of dielectric-metal MetaSurface have been developed with the dielectric layer of the structure was hollow, acting as a microfluidic channel. The electromagnetic fields were strongly confined in the dielectric channel: this significantly enhanced the interaction between the sensing targets and the incident wave. The dielectric constants of sodium chloride and potassium chloride solutions had been determined with various low concentrations [88], achieving high sensitivity (7500 nm/RIU [89]), compared to traditional structures. 

This technique can also be employed to discern different kinds of liquids, such as gasoline [90], liquid paraffin [91], glycerin [92], and water [93].

### 3.3. Surface-Waves for Advanced Medical Diagnostics

MetaSurface sensors attracted large interest in biomedical applications, thanks to the signal amplification effects caused by surface-waves (surface capacitance *C_s_* [46] in Table 1) [94]. The sensing enhancement can be obtained by using complementary structures to match the absorption peak(s) of the sensing targets and the resonant peak(s) of the MetaSurface sensor. 

This approach is extremely useful for skin cancer disease sensing and blood oxygen saturation detection. Structural modifications (size, shape) of chromophores and pigments produce variations of the skin absorption properties [95]. In [96] the sensor consists of multilayered resonating inclusions arranged in a planar array configuration. The possibility to tune the sensing structure resonances with such spectral characteristics allows us to identify several specific diseases.

Another example on how to apply the method described in formulas (1)–(4), is the possibility to detect oxygen levels in the bloodstream. Hemoglobin is responsible for transporting oxygen, carried by human blood, to the several organs of the body, where the oxygen can be used by other cells [97]. The absorption spectra of oxyhemoglobin (HbO2) and deoxyhemoglobin (Hb) are much different. This difference is used for measurements of the amount of oxygen in patient’s blood. In [98] a MetaSurface has been designed to evaluate the ratio HbO2 to Hb, a crucial medical parameter in the study of several pathological diseases, Figure 3d.

Furthermore, flexible MetaSurfaces have been theoretically investigated and fabricated [99], which consisted of a planar array of concentric ring resonators for cancer detection. All such works were of great importance in developing cheap, label-free, real-time, and in-situ sensors (450 nm/RIU [100]). 

Of interest is also the applications developed in the field of protein and DNA detection [101,102]. Small amounts of horseradish peroxidase detection have been demonstrated using the MetaSurface sensor in [103]. A significant shift of the resonant peak occurred when presenting a high sensitivity (600 nm/RIU [104]) for femtomole level of sensed targets. Planar wallpaper MetaSurface (hexagonal and square unit cells) were also utilized for protein detection such as biotin [105] and bovine serum albumin (BSA) molecules [106]. 

A similar approach was used for a label-free biosensing platform for molecular binding detection in living body [107], showing that the frequency shift and attenuation of the transmission coefficient were depended on the bonding amount of sensing targets [108]. The work is crucial to develop sensors able to detect small amount of DNA molecules [109], where traditional free-space measurement approaches could not recognize small DNA optical properties, due to the low absorption of such targets. MetaSurfaces provided an enhanced sensitivity (5 μg/mL [110]) to the tiny refractive index difference between single- and double-stranded DNA for extremely low concentrations, showing a potential approach to the analysis of biologically DNA samples.

## 4. Conclusions

A modeling and design approach to realize MetaSurface sensor has been presented. The proposed method allows us to obtain a full control of the sensor response (amplitude, phase, and bandwidth), and at the same time manipulate at will its features (materials, geometry, and dimensions).

This method has been applied to develop devices for advanced sensing and medical diagnostic applications, showing great performances in terms of sensitivity and selectivity. The technique is very versatile and permits to fully control, manipulate, and tailor the sensors properties. Moreover, the outputs of this method can be used to practically realize the desired sensing platform. Most importantly, the theory here developed can be applied for other applications, beyond sensing and diagnostics.

## Figures and Tables

**Table 1:** Equivalent circuit model elements (first row); series and parallel impedances (second row); capacitive terms of C_tot_: C_f_ the fringing capacitance (electric fields across the narrow neighboring elements of the unit-cell); C_s_ the surface capacitance (charges along the ring surface); and C_g_ the gap capacitance (parallel plate effect) (third row); inductive terms of L_tot_: L_self_ self-inductance of the magnetic loop (currents circulate along the metallic ring); and *M* the mutual inductance between the arms of the same ring (inductive effects among adjacent metallic bars of the unit-cell).

Equivalent Circuit Model Elements		
Impedance	Series	$Z_{series}=\frac{1-\omega^{2}L_{tot}C_{tot}}{j\omega C_{tot}}$
	Parallel	$Z_{parallel}=\frac{j\omega L_{tot}}{1-\omega^{2}L_{tot}C_{tot}}$
Capacitance C_tot_	Gap capacitance *C_g_* [44]	$C_{g}(w,g,t)=\varepsilon_{sample}\frac{wt}{g}$
	Fringing capacitance *C_f_* [45]	$C_{f}(w,g)=\varepsilon_{0}\varepsilon_{sample}\frac{2w+\sqrt{2}g}{\pi }\cosh^{-1}\left(\frac{2w+g}{g}\right)$
	Surface capacitance *C_s_* [46]	$C_{s}(l,w,g,t)=2\varepsilon_{0}\varepsilon_{sample}\frac{t+w}{\pi }\log\left(\frac{8l}{\pi g}\right)$
Inductance L_tot_	Self-inductance *L_self_* [47]	$L_{self}(l,w,t)=\mu_{0}l\left(\frac{1}{2}-\frac{1}{5}\log\left(\frac{w}{t}\right)\right)$
	Mutual inductance *M* [48]	$M(l,w,g,t)=\mu_{0}\frac{1}{4\pi }\left[2l\sinh^{-1}\left(\frac{l}{g}\right)+2\left(l-w-\sqrt{g^{2}+l^{2}}\right)\right]$

**Table 2:** Equivalent circuit model elements for the thickness effect (first row); Substrate Capacitance; (second raw) Additional Capacitance; (third raw) Additional Inductance. Where ω_p_ = 2πf_p_ is the plasma frequency, ω = 2πf is the frequency and δ is the damping frequency of the material used at the considered frequencies.

Equivalent Circuit Model Elements for the Thickness Effect	
Substrate Capacitance X_c_(ε_sub_) [42]	$\varepsilon_{sub}=1+\frac{2}{\pi }\left(\varepsilon_{r}-1\right)\sinh^{-1}\left[\frac{3}{2}\pi \sqrt{\frac{d}{w+s}}\right]$
Additional Capacitance C_add_ [50]	$C_{add}\left(l,w,t\right)=\varepsilon_{0}\varepsilon_{r}\frac{wt}{l}$
Additional Inductance L_add_ [50]	$L_{add}\left(l,w,t\right)=\varepsilon_{0}\frac{l}{wt}\frac{\omega^{2}+\delta^{2}}{\omega^{2}\omega_{p}^{2}}$

**Table 3:** Mutual impedances Z_mutual_ for coupling phenomena. Z_1_ and Z_2_ are the impedances of the unit cell (1) and the adjacent cell (2), respectively. M and C_m_ the mutual inductive and capacitive coupling term, respectively.

The Mutual Impedance Z_mutual_	
Mutual inductance Z_M_	$Z_{M}=Z_{L1}+\frac{\omega^{2}M^{2}}{Z_{L2}}$
Mutual capacitance Z_C_	$Z_{C}=Z_{1}\frac{Z_{2}+C_{m}}{Z_{1}+Z_{2}+C_{m}}$
